# Pancreas divisum and duodenal diverticula as two causes of acute or chronic pancreatitis that should not be overlooked: a case report

**DOI:** 10.1186/1752-1947-2-166

**Published:** 2008-05-19

**Authors:** Massimo De Filippo, Emiliano Giudici, Nicola Sverzellati, Maurizio Zompatori

**Affiliations:** 1 Department of Clinical Sciences, Section of Radiological Sciences, University of Parma, Parma Hospital, Via Gramsci, 43100 Parma, Italy

## Abstract

**Introduction:**

Pancreas divisum is a congenital anatomical anomaly characterized by the lack of fusion of the ventral and dorsal parts of the pancreas during the eighth week of fetal development. This condition is found in 5% to 14% of the general population. In pancreas divisum, the increased incidence of acute and chronic pancreatitis is caused by inadequate drainage of secretions from the body, tail and part of the pancreatic head through an orifice that is too small. The incidence of diverticula in the second part of the duodenum is found in approximately 20% of the population. Compression of the duodenal diverticula at the end of the common bile duct leads to the formation of biliary lithiasis (a principal cause of acute pancreatitis), pain associated with biliary lithiasis owing to compression of the common bile duct (at times with jaundice), and compression of the last part of Wirsung's duct or the hepatopancreatic ampulla (ampulla of Vater) that may lead to both acute and chronic pancreatitis.

**Case presentation:**

We describe the radiological findings of the case of a 75-year-old man with recurrent acute pancreatitis due to a combination of pancreas divisum and duodenal diverticula.

**Conclusion:**

Magnetic resonance cholangiopancreatography is advisable in patients with recurrent pancreatitis (both acute and chronic) since it is the most appropriate noninvasive treatment for the study of the pancreatic system (and the eventual presence of pancreas divisum) and the biliary systems (eventual presence of biliary microlithiasis). Moreover, it can lead to the diagnostic suspicion of duodenal diverticula, which can be confirmed through duodenography with X-ray or computed tomography scan with a radio-opaque contrast agent administered orally.

## Introduction

In the absence of biliary lithiasis or alcohol abuse, pancreas divisum (PD) can be hypothesized as the cause of recurrent or chronic pancreatitis, which may be confirmed through magnetic resonance cholangiopancreatography (MRCP).

Another cause of recurrent or chronic pancreatitis is a diverticulum of the second part of the duodenum. This condition is rarely taken into consideration; when it is small (generally duodenal diverticula (DD) are only a few millimeters in size), it is often missed by radiologists using computed tomography (CT) or magnetic resonance imaging.

A study of the literature showed that there is a surprisingly high incidence of DD in the general population (around 20%). We have only rarely found DD during routine CT and MRCP, and only when they are larger than 3 to 4 cm [1].

A precise etiological diagnosis is fundamental for the treatment of recurrent or chronic pancreatitis: PD and diverticula of the second part of the duodenum are treated in two different ways, the first with endoscopic sphincterotomy of the hepatopancreatic ampulla, the second with surgical removal.

We describe the case of an elderly man with recurrent chronic pancreatitis due to a combination of PD and duodenal diverticulum.

## Case presentation

A 75-year-old man with a clinical history of recurrent pancreatitis (more than two episodes of acute pancreatitis) without risk factors (for example, no previous alcohol abuse, gallstones, hypercalcemia, surgery, use of drugs such as corticosteroids and/or thiazides) was hospitalized for epigastric pain and vomiting.

Clinical examination showed evidence of jaundice. An emergency ultrasound showed lithiasis of the gallbladder, dilation of the main bile duct (9 mm), and a 12 mm hypoechogenic area adjacent to the head of the pancreas. It was initially diagnosed as a cystic lesion of the pancreas.

Laboratory examinations showed an increase in the levels of amylase (306 U/liter, normal 0 to 130 U/liter), lipase (282 U/liter, normal 0 to 58 U/liter), and cholestatic indexes (total bilirubin 3.2 mg/dl, normal 0.1 to 1.1 mg/dl; direct bilirubin 1.4 mg/dl, normal 0.0 to 0.4 mg/dl).

A diagnosis of acute edematous pancreatitis was made. The patient's clinical condition improved significantly after 5 days of pharmacological treatment in hospital with gabexate mesylate, meropenem and omeprazole.

**Figure 1:**
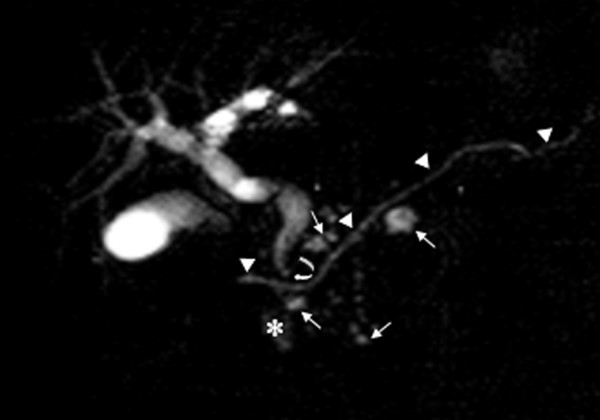
Complete and incomplete pancreas divisum with diverticulum of the second part of the duodenum. The magnetic resonance cholangiopancreatography scan shows the main pancreatic duct (arrowheads) terminates above the distal common bile duct (curved arrow) owing to the presence of pancreas divisum. The multiple small pseudocysts adjacent to Wirsung's duct are markers of recurrent pancreatitis (arrows). Signal irregularity indicated by the asterisk is due to a mixture of fluid and air present inside the duodenal diverticulum (the duodenal 'C' was cancelled by the superparamagnetic contrast medium introduced orally to avoid the overlapping of intestinal fluids with the common bile duct and Wirsung's duct).

**Figure 2:**
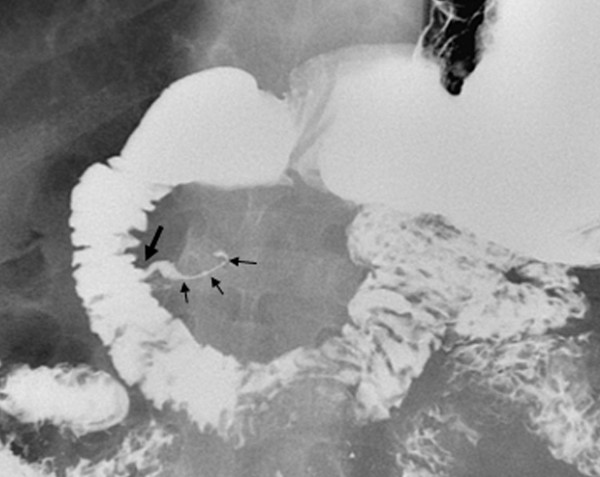
X-ray gastroduodenography with hydrosoluble iodated contrast medium introduced orally showing the diverticulum of the second part of the duodenum. Demonstration of the small neck (large arrow) of the diverticulum. Note that the diverticulum is partially distended by the contrast medium due to the presence of air inside the diverticular lumen (small arrows).

For further investigation, an MRCP, using a 1.5 Tesla unit, was carried out: it revealed evidence of an alithiasic bile duct of normal dimensions with the presence of a 'pancreas divisum' and multiple minute pancreatic pseudocysts (Figure 1). The cystic lesion, evidenced by ultrasonography, was perceived by MRCP as a diverticulum of the second part of the duodenum; this finding was confirmed the following day through radiography with a hydrosoluble iodated contrast medium administered orally (Figure 2).

The patient underwent sphincterotomy of the minor duodenal papilla by means of gastroduodenoscopy to decongest the principal pancreatic duct. Removal of the DD was not carried out owing to clinical recovery. Eight days after the acute event, the patient was discharged in good condition.

## Discussion

PD is a congenital anatomical anomaly characterized by the lack of fusion of the ventral and dorsal parts of the pancreas during the eighth week of fetal development. This condition is found in 5% to 14% of the general population [2].

The major pancreatic duct (Wirsung's duct), in the physiological state and, at rest, has a maximum measurement of 2 mm. It drains the secretions from the head, body and tail of the exocrine pancreas, and ends at the major duodenal papilla (hepatopancreatic ampulla); the accessory pancreatic duct (Santorini's duct) extends through the head of the pancreas, crosses Wirsung's duct and ends at the minor duodenal papilla; both pancreatic outlets are located on the medial wall of the second part of the duodenum at a distance of approximately 10 to 15 mm from each other; the minor papilla are above, the major duodenal papilla below.

In PD, the dorsal pancreatic section drains into the minor duodenal papilla through the major pancreatic duct; the ventral pancreatic duct, the smaller part of the pancreas, merges with the common bile duct at the hepatopancreatic ampulla.

There are two types of PD: complete PD (most common) and incomplete PD (much less common), in which the ventral and dorsal systems remain connected through small-caliber branch ducts [Additional file 1].

In PD, the increased incidence of acute and chronic pancreatitis is caused by inadequate drainage of secretions produced by the body, tail and part of the pancreatic head through an orifice which is too small. The usual therapeutic solution for symptomatic PD is a sphincterotomy of the minor duodenal papilla, which decongests Wirsung's duct [3].

The incidence of diverticula in the duodenum is approximately 20% in the population and is second in frequency to that of the colon; diverticula are formed by the saccular expansion of the mucosal and submucosal layers that together herniate through a defect in the muscular wall as a result of mechanical pressure [1].

The dimensions of DD vary from those of a pea to those of an egg. Singular, or very rarely, multiple DD form frequently in the second part, very infrequently in the third part, and exceptionally in the first part of the duodenum.

Complications of diverticula of the second part of the duodenum are caused by inflammation or ulceration, or may arise from compression of the duodenal wall, the common bile duct or the pancreatic duct due to the close proximity to the engorged and distended diverticula, especially if they are retroduodenal (paravaterian diverticula).

The effects of compression on the end of the common bile duct include the formation of biliary lithiasis (a principal cause of acute pancreatitis), pain associated with biliary lithiasis due to compression of the common bile duct (at times with jaundice), and acute and chronic pancreatitis from compression of the last part of Wirsung's duct or the hepatopancreatic ampulla [1]. The appropriate therapeutic solution for symptomatic DD is surgery.

## Conclusion

The association of PD and DD in the same patient, as in our case, is a rare condition that has not been previously reported in the literature. We believe that it may possibly further increase the incidence of pancreatitis.

It is well known that the principal cause of acute pancreatitis is biliary microlithiasis. It is also true that biliary lithiasis can be determined, as discussed above, by the presence of a DD. Therefore, both PD and DD, and their association, should always be considered in recurrent pancreatitis.

MRCP is advisable in every patient with recurrent pancreatitis, since it is the most appropriate noninvasive treatment for the study of the pancreatic systems (eventual presence of PD) and the biliary systems (eventual presence of biliary microlithiasis) [2].

MRCP, moreover, can lead to the diagnostic suspicion of duodenal diverticula, which can be confirmed through duodenography by X-ray or CT scan with the administration of an orally radio-opaque contrast agent.

## Abbreviations

CT: computed tomography; DD: duodenal diverticula; MRCP: magnetic resonance cholangiopancreatography; PD: pancreas divisum.

## Competing interests

The authors declare that they have no competing interests.

## Authors' contributions

MD and EG collected the data and drafted the manuscript. Both NS and MZ revised and approved the final manuscript.

## Consent

Written informed consent was obtained from the patient for publication of this case report and any accompanying images. A copy of the written consent is available for review by the Editor-in-Chief of this journal.

## Supplementary Material

Additional file 1 Drawings of pancreas divisum and duodenal diverticula. Complete and incomplete pancreas divisum with diverticulum of the second part of the duodenum. (a) Complete; (b) incomplete. Click here for file
