# Supplementary figures and images for: Pancreas divisum and duodenal diverticula as two causes of acute or chronic pancreatitis that should not be overlooked: a case report
Source: J Med Case Rep. 2008 May 19;2:166. doi: 10.1186/1752-1947-2-166 (PMC2413259; doi:10.1186/1752-1947-2-166)

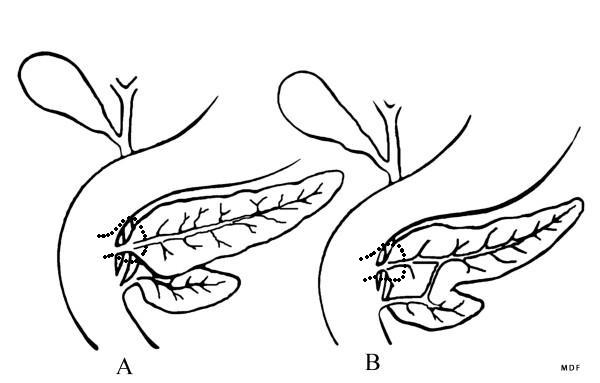

Supplement: Additional file 1 — Drawings of pancreas divisum and duodenal diverticula. Complete and incomplete pancreas divisum with diverticulum of the second part of the duodenum. (a) Complete; (b) incomplete. [file 1752-1947-2-166-S1.jpeg]
